# Supplementary material for: Investigating the Interactions of the Cucumber Mosaic Virus 2b Protein with the Viral 1a Replicase Component and the Cellular RNA Silencing Factor Argonaute 1
Source: Viruses. 2024 Apr 25;16(5):676. doi: 10.3390/v16050676 (PMC11125589; doi:10.3390/v16050676)
Supplement: Supplementary file 1 [file viruses-16-00676-s001.zip › viruses-2943433-supplementary.pdf]

Supplementary File

Investigating the interactions of the cucumber mosaic virus 2b protein with the viral 1a replicase component and the cellular RNA silencing factor Argonaute 1

Sam Crawshaw[1], Alex M. Murphy[1], Pamela J. E. Rowling[2], Daniel Nietlispach[3], Laura S. Itzhaki[2] and John P. Carr[1]*

1. Department of Plant Sciences, University of Cambridge, Downing Street, Cambridge CB2 3EA, United Kingdom

2. Department of Pharmacology, University of Cambridge, Tennis Court Rd, Cambridge CB2 1PD, United Kingdom

3. Department of Biochemistry, University of Cambridge, Sanger Building, 80 Tennis Court Rd, Cambridge CB2 1GA, United Kingdom

*Author for Correspondence: jpc1005@cam.ac.uk

Supplementary Materials

Table S1. Primer sequences.

Primer name	Sequence (5′-3′)
**Gateway cloning**	
2b attB Rv	GGGGACCACTTTGTACAAGAAAGCTGGGTTGAAAGCACCTTCCGCCCA
2b attB Fw	GGGGACAAGTTTGTACAAAAAAGCAGGCTTCATGGAATTGAACGTAGGT
2bΔ1-17 Fw	GGGGACAAGTTTGTACAAAAAAGCAGGCTTCATGGTGGAGGCGAAGAAGC
2bΔ95-110 Rv	GGGGACCACTTTGTACAAGAAAGCTGGGTTAAAATCATGGTCTTCCGCCGA
2bΔ85-110	GGGGACCACTTTGTACAAGAAAGCTGGGTTACGAGAGGCCTCAGACTCG
2bΔ74-110	GGGGACCACTTTGTACAAGAAAGCTGGGTTCACATGGCGGCATGAC
2bΔ56-110	GGGGACCACTTTGTACAAGAAAGCTGGGTTTAGGAAGCGGAATAGTCTGAGATTTGAAC
2bΔ83-110 Rv	GGGGACCACTTTGTACAAGAAAGCTGGGTTTCAGGCCTCAGACTCGGG
2bΔ1-55 Fw	GGGGACAAGTTTGTACAAAAAAGCAGGCTTAATGCCGTTCTATCAAGTGGATGGTTCG
2bΔ61-110 Rv	GGGGACCACTTTGTACAAGAAAGCTGGGTTCACTTGATAGAACGGTAGGAAGCG
2bΔ65-110 Rv	GGGGACCACTTTGTACAAGAAAGCTGGGTTTTCCGAACCATCCACTTGATAGAACG
2bΔ69-110 Rv	GGGGACCACTTTGTACAAGAAAGCTGGGTTTGACCCTGTCAGTTCCGAACC
**Q5 mutagenesis**	
2bLS/Fny(83-93) Rv	GGCCTCAGACTCGGGTAA
2bLS/Fny(83-93) Fw	TTTGACGATACAGATTGGTTCG
2bΔ56-60/65 Rv	TAGGAAGCGGAATAGTCTGAG
2bΔ39-48 Fw	CTCAGACTATTCCGCTTCCTAC
2bΔ39-48 Rv	GTGACCTCGTTCCCGTCG
2bΔ56-65 Fw	ACAGGGTCATGCCGC
2bΔ56-60 Fw	GATGGTTCGGAACTGACAG
2b56aaa65 Rv	CGCCGCCGCCGCCGCCGCCGCCGCCGCCGCTAGGAAGCGGAATAGTCTGAG
2b56aaa60 Rv	CGCCGCCGCCGCCGCTAGGAAGCGGAATAGTCTGAG
2bFny/LS(56-60) Fw	CCGTTCCATGGAGTGGATGGTTCGGAA
2bΔ83-93 (step 2) Rv	GAAAGCACCTTCCGCCCATTCGTTACCGGCGAACCAATCTGTATCGTCAAAGGCCTC
2bΔ83-93 (step 1) Rv	CCAATCTGTATCGTCAAAGGCCTCAGACTCGGGTAAC
2b Fw	ATGGAATTGAACGTAGGTGCAATGAC

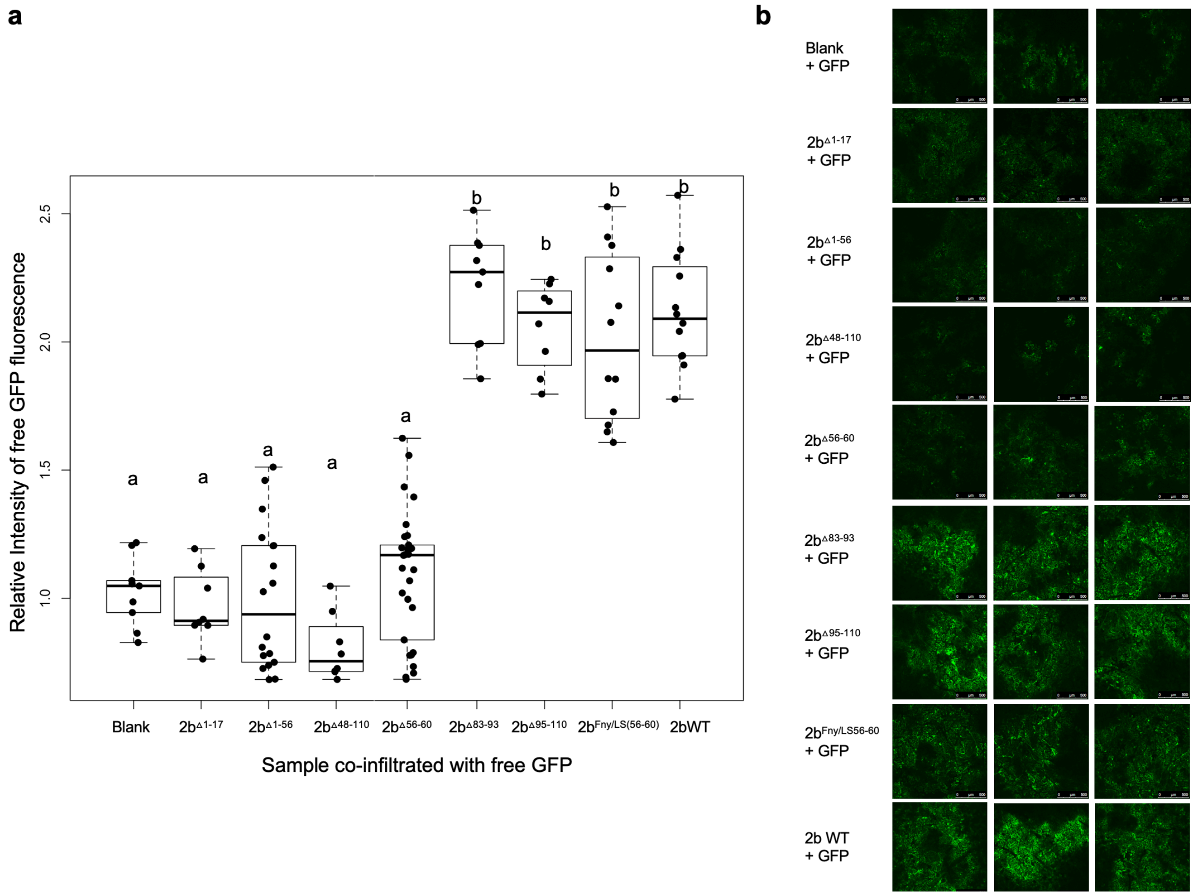

Figure S1. Mutations in the C-terminal region of the CMV 2b protein do not impact its RNA silencing suppressor activity. Green fluorescent protein (GFP) was expressed transiently, under a 35S promoter, in agroinfiltrated leaves of *Nicotiana benthamiana*. **a.** The relative intensity of GFP fluorescence was quantified using ImageJ as the integrated density (IntDen) of each image, for each treatment 12 days after infiltration. Individual relative fluorescence values are presented as jitter plots with each mean value and standard error depicted as black bars. Compared to the intensity of fluorescence emitted by leaves expressing GFP only, the relative intensity values of GFP fluorescence emitted by leaf tissue agroinfiltrated with mixtures of *A. tumefaciens* cells that included those carrying constructs expressing the full length 2b protein (WT), 95-110, 83-93 or Fny/LS(56-60) mutant versions of the 2b protein were significantly greater. Lower case letters *a* and *b* indicate mean values for relative fluorescence intensity that are significantly different from each (P <0.0001: Tukey's multiple comparison of means). Values labelled with the same letter are not significantly different from each other. Number of independent leaves imaged for each treatment, n > 6. **b.** Typical confocal images of GFP fluorescence in the presence of full length or mutant versions of the CMV 2b protein as indicated.

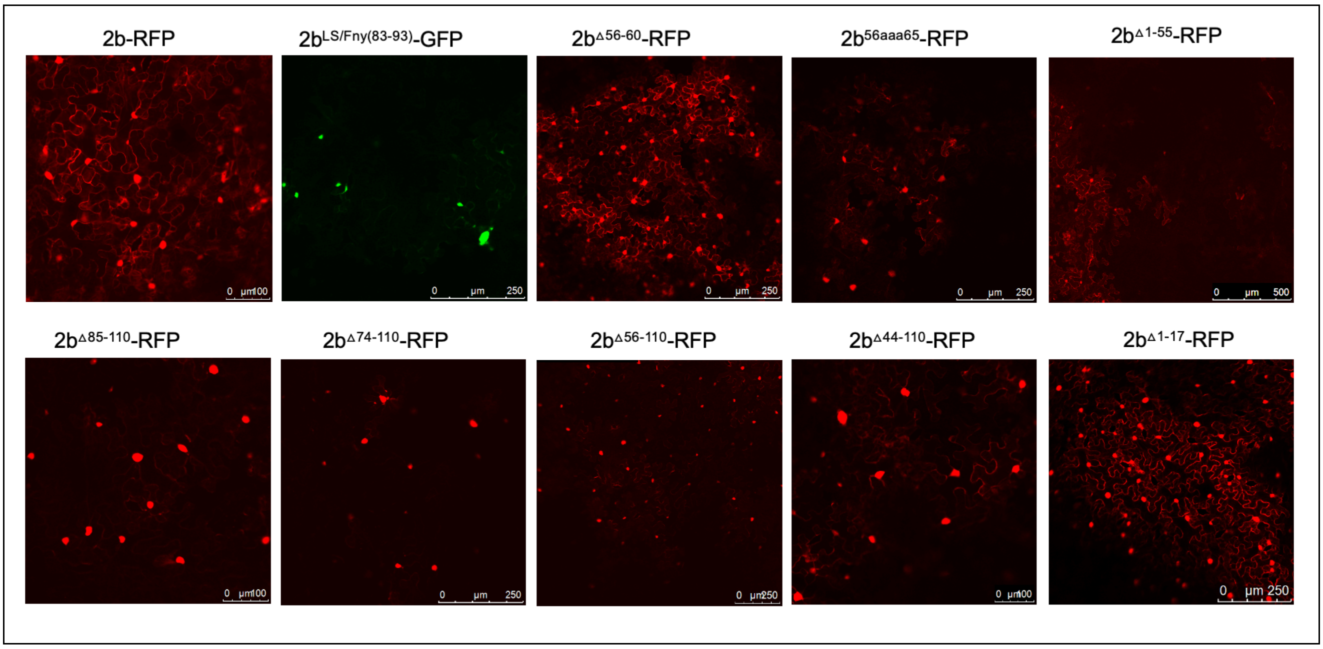

Figure S2. Mutations affecting the subcellular localization of the cucumber mosaic virus 2b protein. Agroinfiltration was used for transient expression in *N. benthamiana* leaves of C-terminal red fluorescent protein 2b-(RFP) or C-terminal green fluorescent protein 2b-(GFP) fusion protein. All mutant versions of the 2b protein showed nuclear and cytoplasmic localization. However, mutant versions of the 2b protein lacking residues at their C-terminus (such as 85-110, 74-110, 56-110 or 44-110) showed an increased nuclear localization compared to the wild-type protein. Increased nuclear localization was also seen for the LS/Fny chimeric mutant in which residues present in the Fny-CMV 2b protein (residues 83-93) but absent in the sequence of the LS-CMV 2b protein, were introduced into a chimeric LS-CMV protein. However, the N-terminal mutant from residues 1-17, mutations from 56-60 or substitution of alanine from 56-60 had no impact on the localization of CMV 2b. In contrast, deletion of residues from 1-55 caused a reduction in nuclear localization and an increase in cytoplasmic localization.

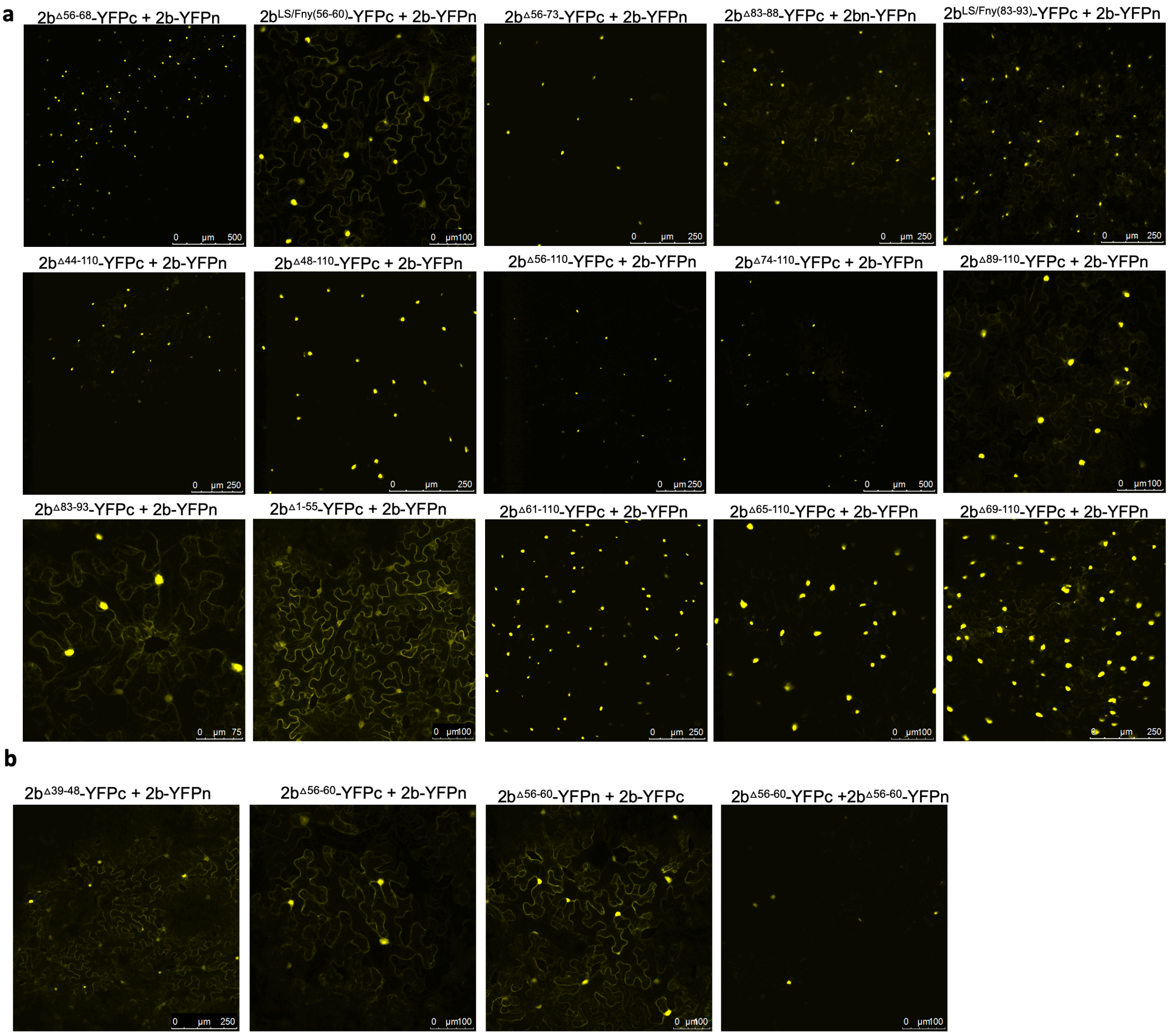

Figure S3. Mutations affecting the ability of the 2b protein to self-interact. Bimolecular fluorescence complementation was used to compare the self-interaction properties of the 2b protein of Fny-CMV with mutant versions of the 2b protein using fusion proteins with the N- and C-terminal domains of the yellow fluorescent protein (2b-YFPn and 2b-YFPc). **a.** All mutant proteins were able to form heterodimers with WT 2b protein *in vivo*, indicated by yellow fluorescence. The intracellular distributions of these homodimers were consistent with those seen for 2b-RFP or 2b-GFP mutant proteins and show a greater proportion of some mutant 2b proteins being present in the nucleus. **b.** Tagged mutant versions of the 2b protein lacking residues between 56-60 interacted strongly with tagged full length WT 2b protein. However, self-interaction with two mutant versions of the 2b protein resulted in markedly reduced levels of self-interaction.

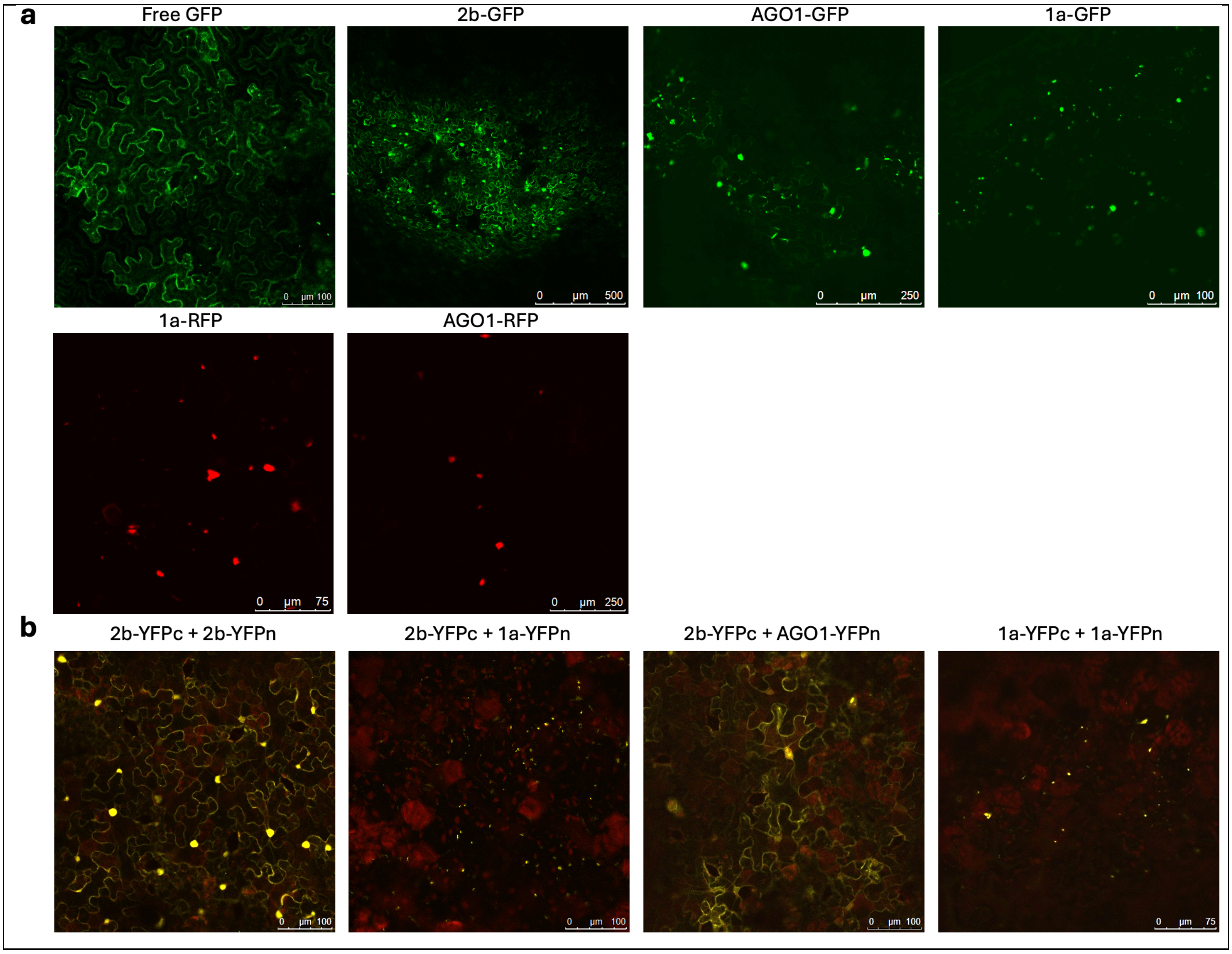

Figure S4. Subcellular distribution of fluorescent proteins. Agroinfiltration was used for transient expression in *N. benthamiana* leaves of red fluorescent protein (RFP), green fluorescent protein (GFP) or split yellow fluorescent protein (YFP) fusions. **a.** The subcellular localisation of fluorescently tagged Fny-CMV 2b, Fny-CMV 1a and AGO1 proteins are shown in comparison to the subcellular distribution of free GFP. **b.** Bimolecular fluorescence complementation was used to compare protein-protein interactions between Fny-CMV 2b, Fny-CMV 1a and AGO1 proteins using fusion proteins with the N- and C-terminal domains of the yellow fluorescent protein. Images are shown with a chlorophyll background.

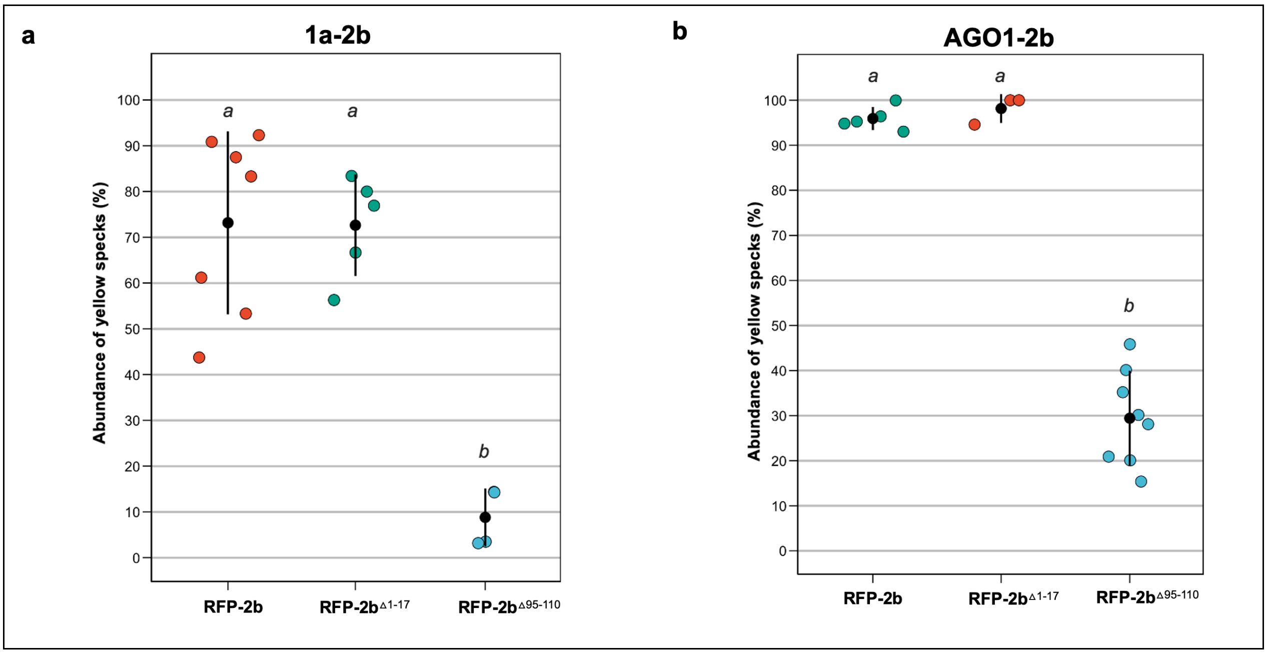

Figure S5. The C-terminal domain of the cucumber mosaic virus 2b protein enhances its interaction with the Fny-CMV 1a and AGO1 proteins. Red fluorescent protein (RFP)-tagged CMV 2b proteins containing deletions in their N-terminal domain (RFP-$2b^{\Delta 1\text{-}17}$) or C-terminal domain (RFP-$2b^{\Delta 95\text{-}110}$) were transiently co-expressed with green fluorescent protein (GFP)-tagged Fny-CMV 1a or AGO1 proteins in agroinfiltrated leaves of *N. benthamiana*. The abundance of yellow specks in each image was quantified by calculating the number of yellow specks as a percentage of the total specks present in the image. Individual abundance values are presented as jitter plots with each mean value and standard error depicted as black bars. Since yellow specks result from the overlay of GFP and RFP fluorescent signals they were used as a measure of co-localization between co-infiltrated GFP- and RFP-tagged proteins. **a** Co-localization results for 1a-GFP with RFP-$2b^{\Delta 95\text{-}110}$, RFP-$2b^{\Delta 1\text{-}17}$ and RFP-2b. Lower case letters *a* and *b* indicate mean values for abundance of yellow specks that are significantly different from each other ($P < 0.0001$: Tukey's multiple comparison of means). Values labelled with the same letter are not significantly different form each other. Deletion of the N-terminal domain of the 2b protein had no significant effect on colocalization with the CMV 1a protein. However, deletion of the C-terminal domain of the 2b protein significantly reduced colocalization with the CMV 1a protein. **b** Co-localization results for AGO1-GFP with RFP-$2b^{\Delta 95\text{-}110}$, RFP-$2b^{\Delta 1\text{-}17}$ and RFP- 2b. Lower case letters *a* and *b* indicate mean values for abundance of yellow specks that are significantly different from each other ($P < 0.0001$: Tukey's multiple comparison of means). Values labelled with the same letter are not significantly different form each other. Deletion of the N-terminal domain of 2b had no significant effect on colocalization with AGO1. However, deletion of the C-terminal domain of 2b significantly reduced colocalization with AGO1. Number of independent leaves imaged for each treatment, $n \geq 3$.

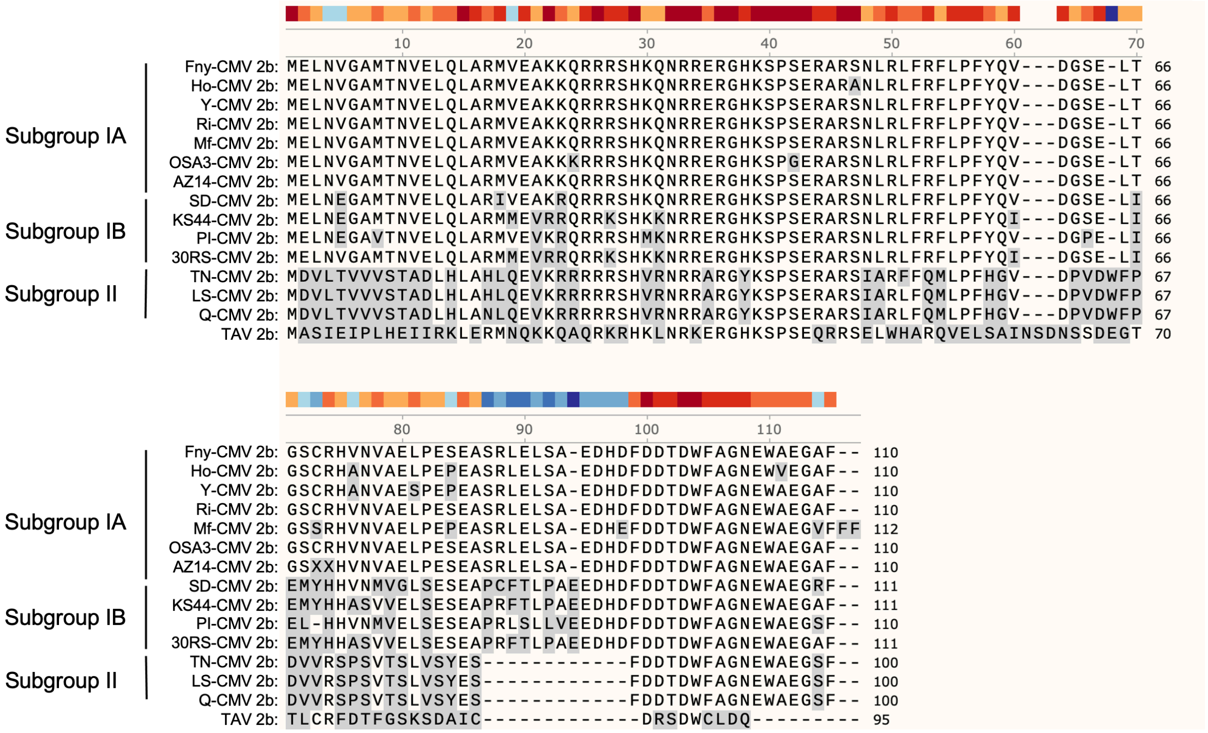

Figure S6. The amino acid sequences of 2b protein orthologues encoded by a selection of strains of cucumber mosaic virus (CMV) from Subgroups IA, IB and II and the 2b protein from tomato aspermy virus (TAV). The Fny-CMV 2b protein sequence is given as a reference to compare with 2b protein sequences from a small number of other CMV strains to highlight certain differences and similarities between 2b proteins encoded by viruses in Subgroups IA, IB and II. Differences in 2b protein primary sequences between the Fny-CMV and other strains are highlighted in gray. The numbering of amino acid residues is based on the Fny-CMV 2b protein sequence. The GenBank accession numbers for the sequences used in this alignment are NC002035 for Fny-CMV (2b-Fny), D12538 for Y-CMV (2b-Y), LC593245 for Ho- CMV (2b- Ho), AM183118 for RI-8-CMV (2b-RI-8), AJ276480 for Mf-CMV (2b-Mf), HE971489 for OSA3-CMV (2b-OSA3), QBH72281 for AZ14-CMV (2b-AZ14), D86330 for SD-CMV, CBG76802 for KS44-CMV (2b-KS44), CAJ65577 for PI-1-CMV (2b-PI-1), FN552601 for 30RS-CMV (2b-30RS), BAD15371 for TN-CMV (2b-TN), AF416900 for LS-CMV (2b- LS), Q66125 for Q-CMV (2b-Q) and AJ320274 for KC-TAV (TAV 2b).

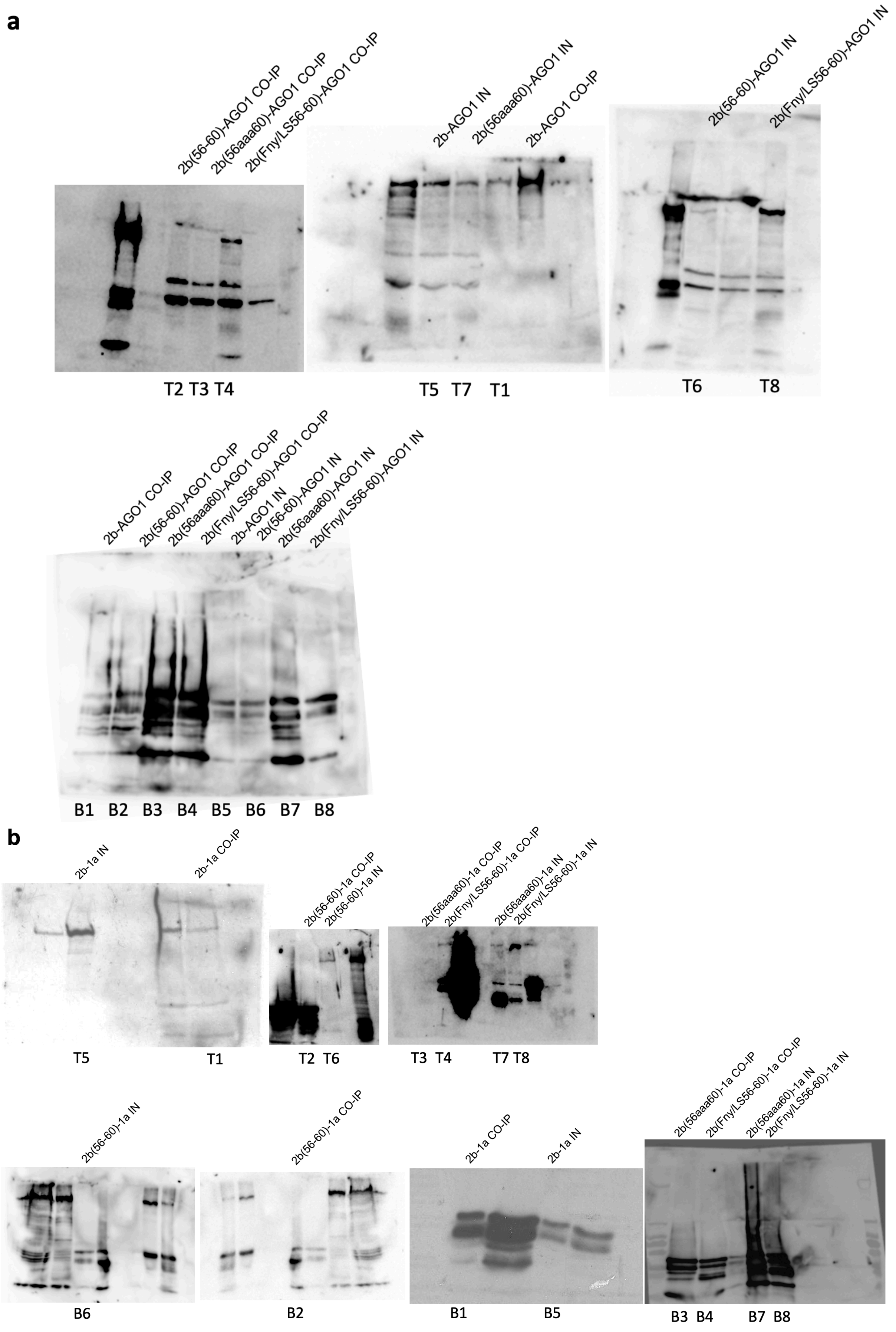

Figure S7. Whole gel images showing association of wild type and mutant Fny-CMV 2b proteins with Fny-CMV 1a or AGO1 proteins demonstrated by co-immunoprecipitation. **a.** RFP- tagged 2b proteins were co-expressed with

GFP tagged AGO1 in *N. benthamiana* leaves. Total protein was subjected to immunoprecipitation with GFP-Trap or RFP-Trap beads followed by immunoblot analysis with anti-RFP antibodies to detect 2b-RFP or anti-GFP antibodies to detect AGO1-GFP. Bands are labelled with the contents of the total protein extract and treatment: input sample (IN), Co-immunoprecipitation with interacting partner (CO-IP). The position where each band appears in the composite blot figures is denoted by T or B referring to the top or bottom row of gel strips. **b.** RFP-tagged 2b proteins were co-expressed with GFP tagged 1a protein in *N. benthamiana* leaves. Total protein was subjected to immunoprecipitation with GFP-Trap or RFP-Trap beads followed by immunoblot analysis with anti-RFP antibodies to detect 2b-RFP or anti-GFP antibodies to detect 1a-GFP. Bands are labelled with the contents of the total protein extract and treatment: input sample (IN) or Co-immunoprecipitation with interacting partner (CO-IP). The position where each band appears in the composite blot figures is denoted by T or B referring to the top or bottom row of gel strips.

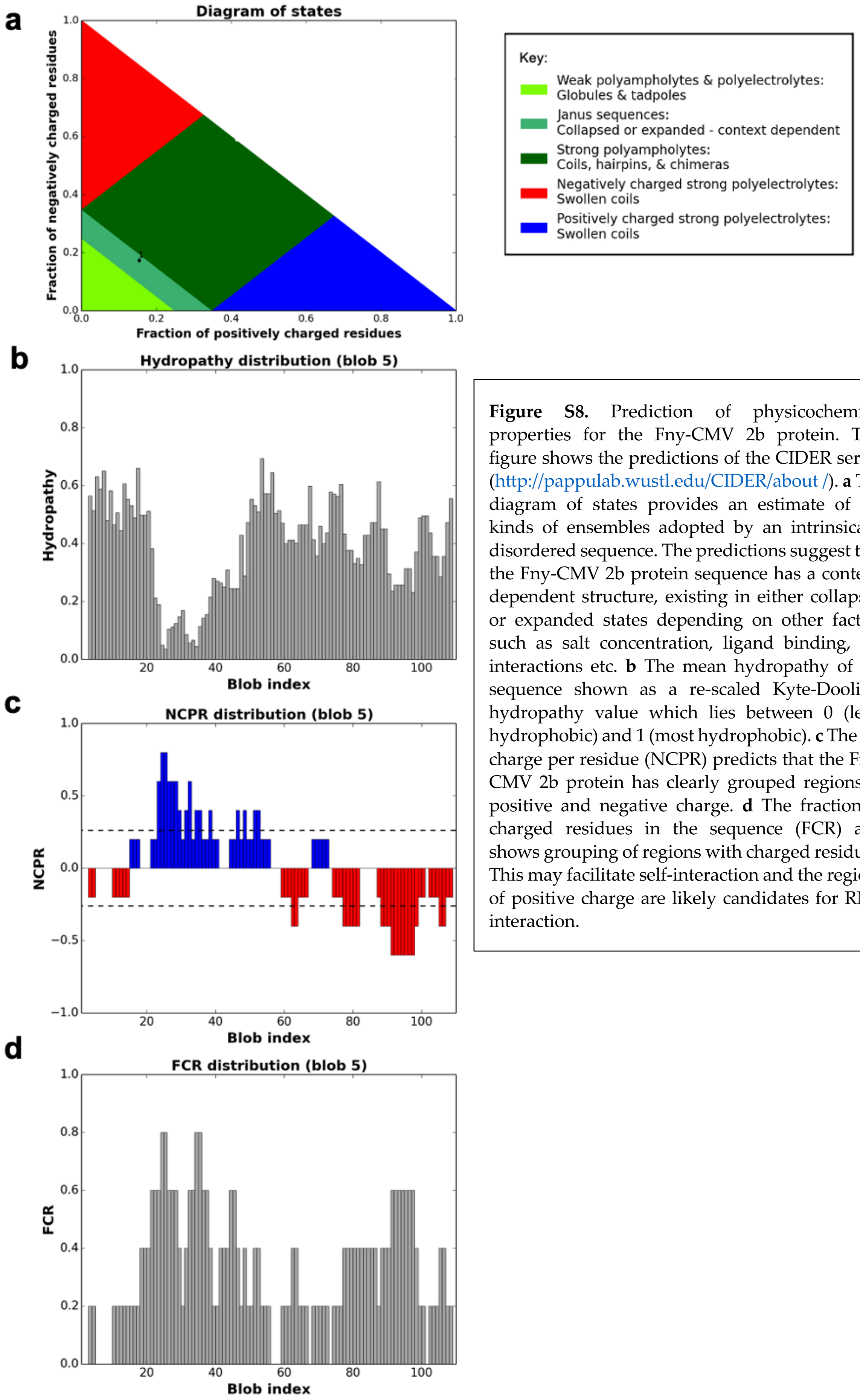

Figure S8. Prediction of physicochemical properties for the Fny-CMV 2b protein. This figure shows the predictions of the CIDER server (http://pappulab.wustl.edu/CIDER/about /). **a** The diagram of states provides an estimate of the kinds of ensembles adopted by an intrinsically disordered sequence. The predictions suggest that the Fny-CMV 2b protein sequence has a context-dependent structure, existing in either collapsed or expanded states depending on other factors such as salt concentration, ligand binding, *cis*-interactions etc. **b** The mean hydropathy of the sequence shown as a re-scaled Kyte-Doolittle hydropathy value which lies between 0 (least hydrophobic) and 1 (most hydrophobic). **c** The net charge per residue (NCPR) predicts that the Fny-CMV 2b protein has clearly grouped regions of positive and negative charge. **d** The fraction of charged residues in the sequence (FCR) also shows grouping of regions with charged residues. This may facilitate self-interaction and the regions of positive charge are likely candidates for RNA interaction.

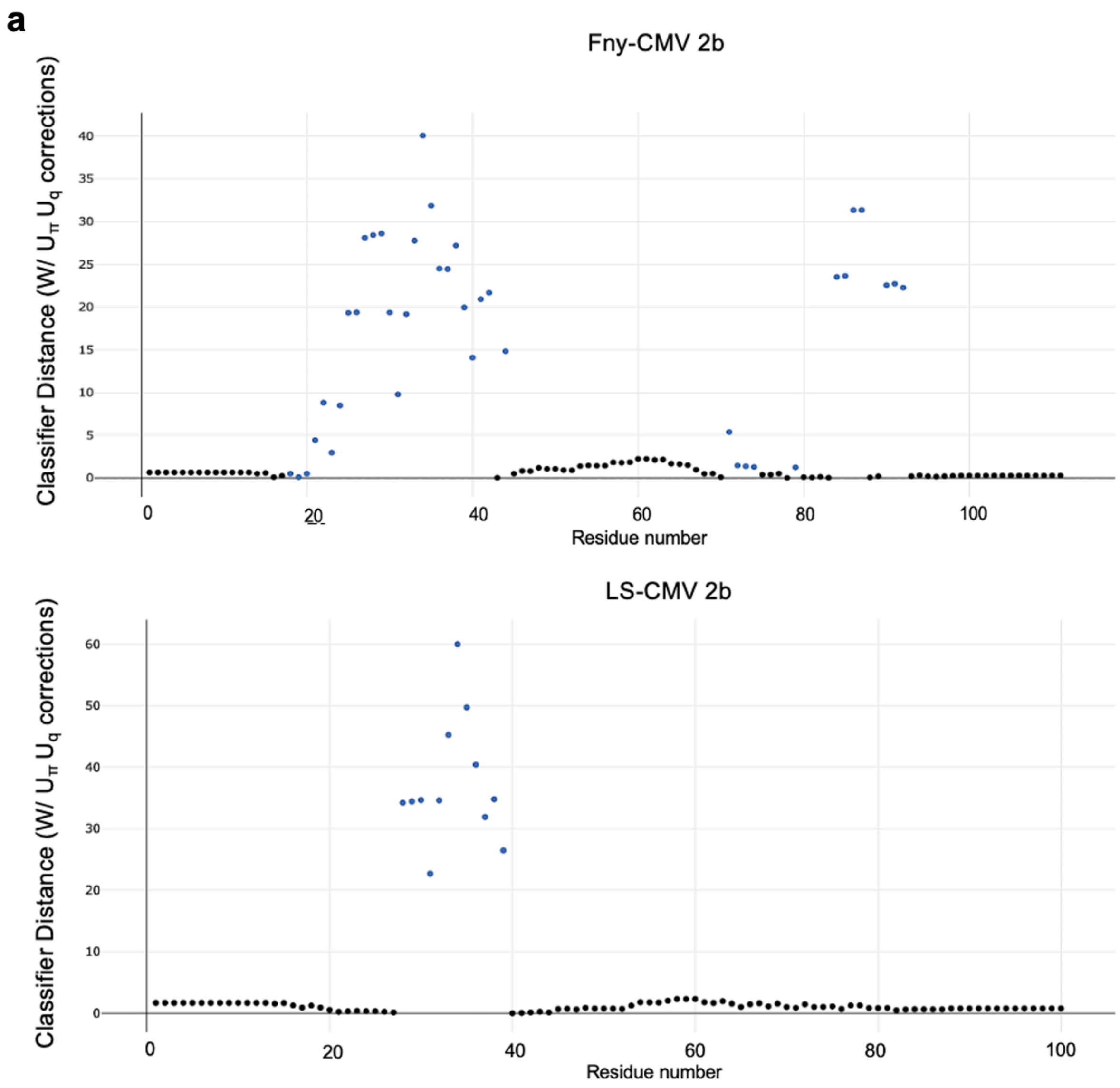
a
Fny-CMV 2b
Classifier Distance (W/ U_π U_q corrections)
0
5
10
15
20
25
30
35
40
0
20
40
60
80
100
Residue number
LS-CMV 2b
Classifier Distance (W/ U_π U_q corrections)
0
10
20
30
40
50
60
0
20
40
60
80
100
Residue number

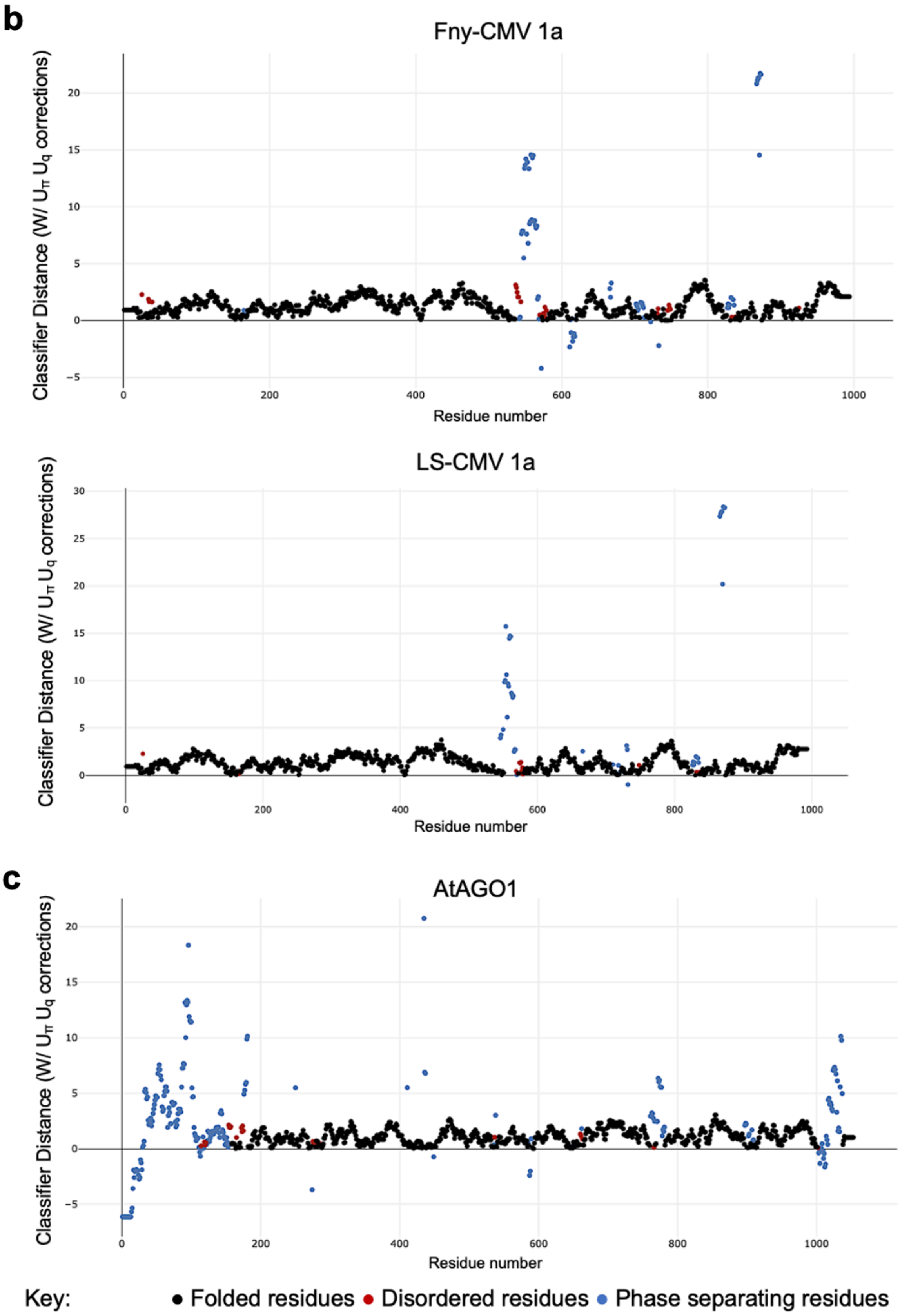

Figure S9. Predictions of phase separation. The ParSe v2 program identifies protein regions likely to exhibit physiological phase separation behavior. The results shown here are for the modified ParSe v2 program accounting for the effects of interactions between amino acids (termed U_{π} for π–π and cation–π interactions and U_q for charge-based effects). Residues are color coded based on their predicted state with black representing folded residues, red representing disordered residues and blue representing phase separating residues. The classifier

distance on the Y-axis represents the confidence in the assigned states with higher values relating to higher confidence. **a** The Fny-CMV 2b protein is predicted to contain two regions which promote phase separation behavior while the LS-CMV 2b protein contains only one such region. **b** The Fny-CMV 1a protein contains residues which are likely to promote phase separating behavior, but these represent a smaller percentage of residues than those seen in the Fny-CMV 2b protein. The LS-CMV 1a protein also contains residues which are likely to promote phase separation, but there are slightly fewer such residues than in the Fny-CMV 1a protein (41 compared with 66). **C,** The *A. thaliana* AGO1 protein is also predicted to contain residues promoting phase separation with the greatest density at its N-terminus.

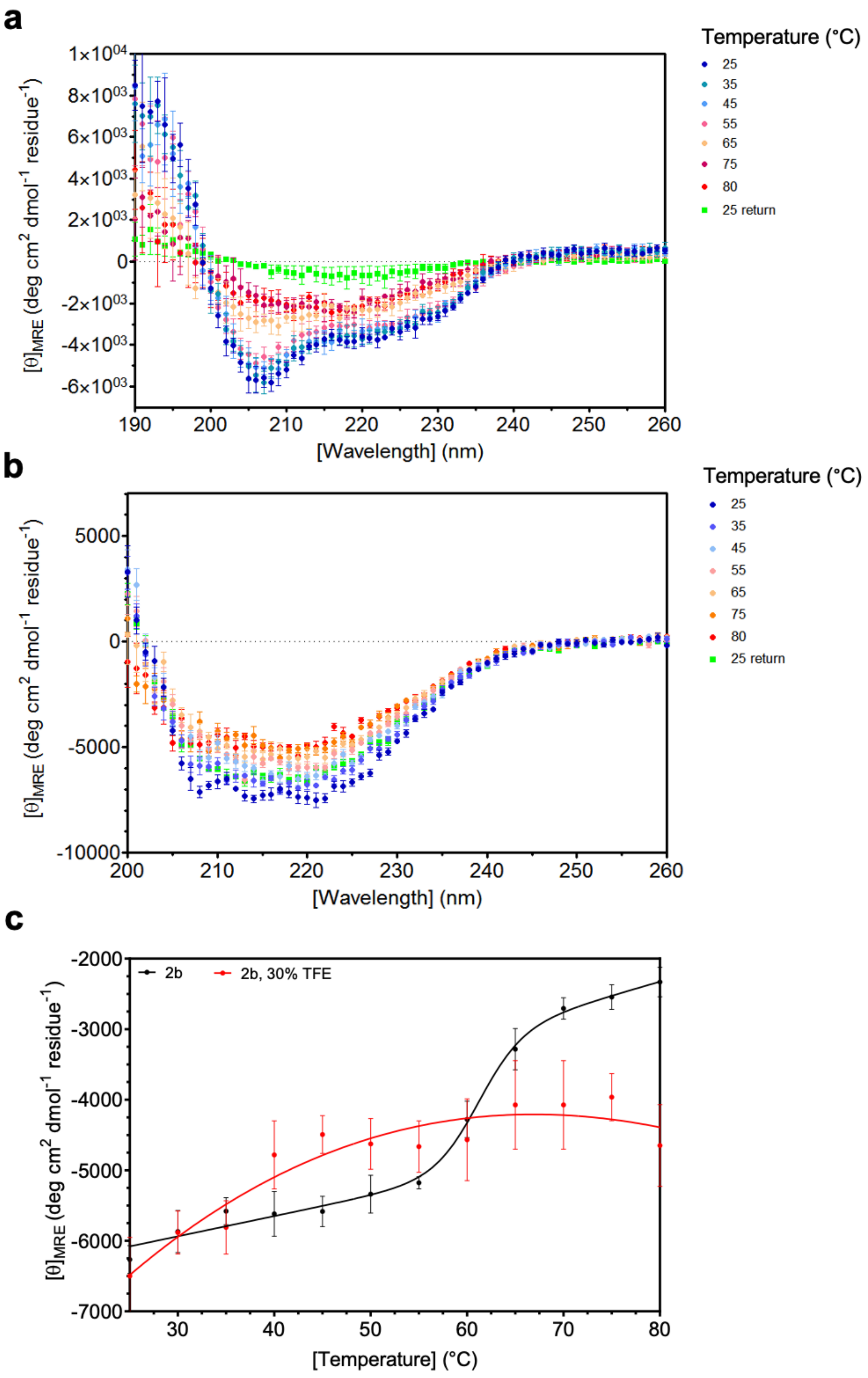
a
Temperature (°C)
25
35
45
55
65
75
80
25 return
[θ]MRE (deg cm2 dmol-1 residue-1)
[Wavelength] (nm)
b
Temperature (°C)
25
35
45
55
65
75
80
25 return
[θ]MRE (deg cm2 dmol-1 residue-1)
[Wavelength] (nm)
c
2b
2b, 30% TFE
[θ]MRE (deg cm2 dmol-1 residue-1)
[Temperature] (°C)

Figure S10. Temperature-induced Denaturation of the Fny-CMV 2b protein. Circular dichroism data were obtained at a protein concentration of 13.5 μM 2b in 10 mM phosphate buffer pH 7.5 at temperatures ranging from 25 °C to 80 °C. **a** Following heating, the 2b protein is irreversibly denatured as cooling (green symbols) does not revert the protein to its initial state at 25 °C (dark blue symbols). **b** Following the addition of 30% trifluroethanol (α -helical structure in proteins) the 2b protein does not completely unfold even when heated to 80 °C and following cooling back to 25 °C the protein does revert to its initial folded state. **c** There is a transition between folded and unfolded state where 50% of the 2b protein is in each state at 61 °C when fitted to a Boltzmann model with sloping baselines (black symbols) measured at 208 nm. All results are shown as the mean of five scans ± SEM.

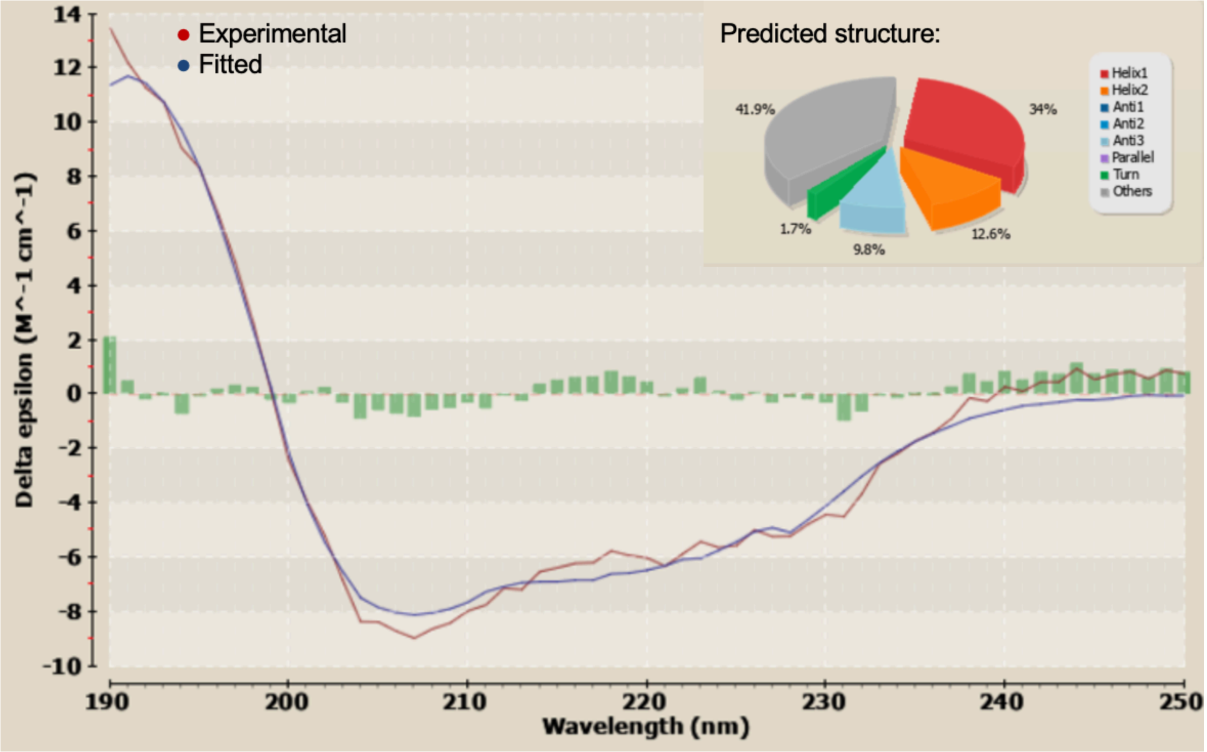

Figure S11. Alternate analysis of circular dichroism data. The circular dichroism data shown in fig. 9 for Fny-CMV 2b protein in the absence of TFE was also run through the BeStSel program (v1.3.230210). This program predicted a similar level of disorder within the 2b sequence (41.9 %) but a larger percentage of α-helical residues (46.6 %) and a smaller percentage of β sheet (9.8 %). This program does not use datasets specifically trained on disordered sequences, but the results do align more closely with the structural data obtained from NMR analysis.

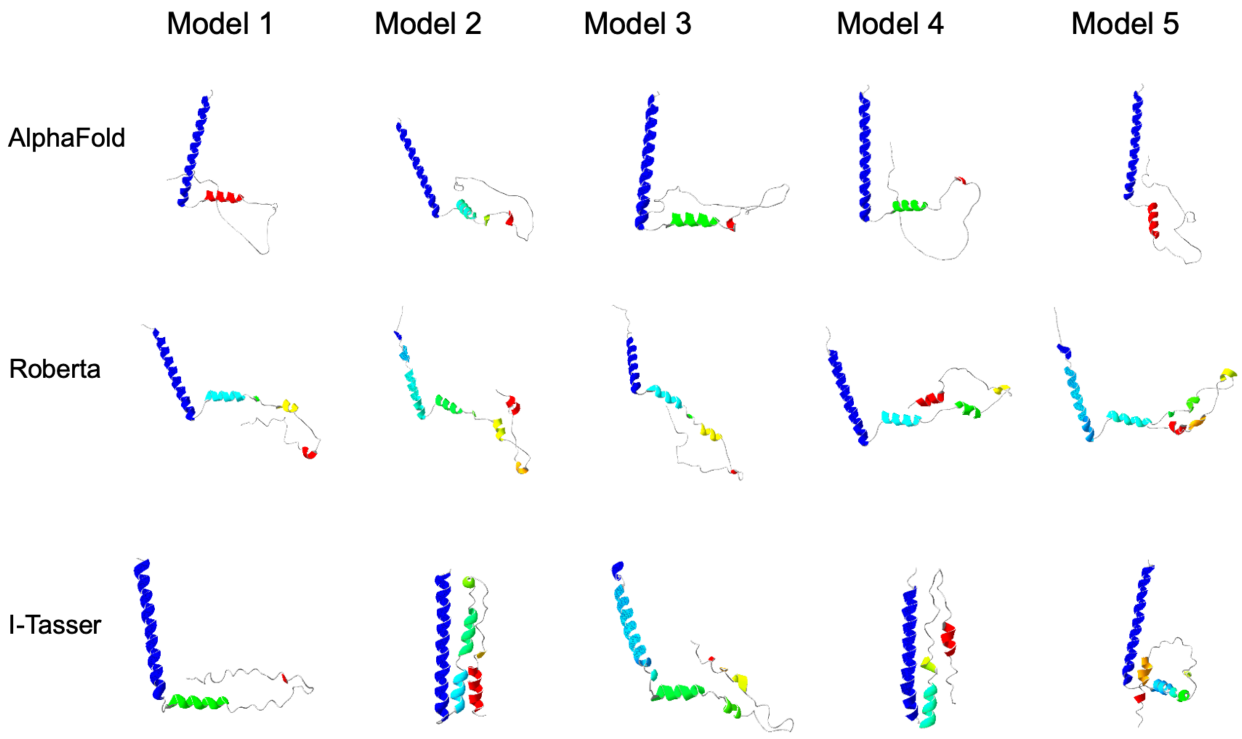

Figure S12. Polypeptide folding predictions for the Fny-CMV 2b protein. Modelling predictions are shown from Alpha-Fold 2, RoseTTA fold and I-Tasser. All models show two α-helices in the N-terminal half of the 2b protein and the majority of models have a largely disordered C-terminal half.
